# The exhaustion of lymphocytes is the main factor that decreases the sensitivity of QFT-GIT detection in silicosis

**DOI:** 10.1186/s12865-022-00538-9

**Published:** 2022-12-31

**Authors:** Xin Yu, Li-Na Huang, Jun-Chi Xu, Yi-Yan Song, Hui Chen, Cui-Lin Shi, Pei-Jun Tang, Tao Tao, Ye-Han Zhu

**Affiliations:** 1 Department of Pulmonary and Critical Care Medicine, The First Affiliated Hospital of Soochow University, 899, Pinghai Road, Jiangsu 215000 Suzhou, People’s Republic of China; 2 Department of Clinical Laboratory, The Fifth People’s Hospital of Suzhou, 10, Guanqian Road, Suzhou, Jiangsu 215000 People’s Republic of China

**Keywords:** QFT-GIT, Silicosis, Tuberculosis, PD-1

## Abstract

**Background:**

Tuberculosis infection is a major complication of silicosis, but there is no study on whether silicosis can affect the sensitivity of QuantiFERON-TB Gold In-Tube (QFT-GIT) assays. This study will analyze the relationship between silicosis and QFT-GIT, determine the main factor of the QFT-GIT sensitivity decrease in silicosis and explore the methods to increase the sensitivity.

**Methods:**

Silicosis patients with positive tubercle bacillus cultures were collected. The QFT-GIT, flow cytometry and blocking antibodies were used.

**Results:**

The sensitivity of QFT-GIT in silicosis patients (58.46%) was significantly decreased and the expression of PD-1 on T cells and CD56^+^NK cells in pulmonary tuberculosis combined with silicosis were higher than normal tuberculosis patients and silicosis only patients. Further analysis found that the ratio of PD-1^+^CD4^+^T and IFN-γwere negatively correlated and blockaded the PD-1 pathway with antibodies can restore the sensitivity of QFT-GIT in silicosis.

**Conclusions:**

This is the first study to analyze the relationship between immune exhaustion and QFT-GIT in silicosis and found that the sensitivity of QFT-GIT was decreased by the expression of PD-1 on lymphocytes. Antibody blocking experiments increased the expression of IFN-γ and provided a new method to improve the sensitivity of QFT in silicosis. The study also found that silicosis can increase PD-1 expression. As PD-1 functions in infectious diseases, it will promote immune exhaustion in silicosis and lead to tuberculosis from latent to active infection. The study provided theoretical evidence for the diagnosis and immunotherapy of silicosis complications, and it has great value in clinical diagnostics and treatment.

## Introduction

Tuberculosis is a common and serious complication of silicosis. The probability of concurrent pulmonary tuberculosis at the time of phase I–II disease is 10–30%. When silicosis enters phase III, tuberculosis occurs in 50–90% of patients, and 45% of silicosis patients die directly from tuberculosis [1–3]. Studies have found that with the development of silicosis, the incidence of pulmonary tuberculosis increases [2]. At the same time, when silicosis is complicated by pulmonary tuberculosis, the two diseases promote each other and accelerate deterioration, so timely detection of tuberculosis infection and targeted treatment are particularly important for patients with silicosis [2, 3].

The IFN-γ release in vitro test (TB-IGRA) is a novel detection method for the early diagnosis of clinical pulmonary tuberculosis. It is an in vitro diagnostic method that tests the release of IFN-γ to help diagnose tuberculosis or determine whether the body is infected with mycobacterium tuberculosis. The principle is that after mycobacterium tuberculosis infection, the body can produce antigen-specific T cells, and when the body is stimulated by mycobacterium tuberculosis antigen again, the antigen-specific T cells can be quickly activated and proliferate, releasing IFN-γ and thereby aiding in the diagnosis of tuberculosis [4]. Compared with traditional detection methods, TB-IGRA has several positive characteristics, such as rapidity, high sensitivity and high specificity, so it is accepted by most of laboratories. Since TB-IGRA is a detection method based on the host's immune response to mycobacterium tuberculosis, the results of TB-IGRA are closely related to the patient's immune status [4]. Existing studies have confirmed that compared with other tests (TST, X-ray and culture), TB-IGRA is considered to be the most sensitive method of tuberculosis diagnostics [5]. Of the two commercially available TB-IGRA, QuantiFERON-TB Gold (QFT-GIT) assays are more commonly used in routine clinical practice than T-SPOT.TB assays. Some research has compared the performance between TST and TB-IGRA in silicosis complicated by tuberculosis and found that TB-IGRA is more suitable for tuberculosis diagnostics. However, there has been no study on whether silicosis can affect the sensitivity of TB-IGRA [6, 7]. Therefore, the study systematically analyzed the effect of silicosis on QFT-GIT assays and explored the causes of and solutions.

## Methods

### Patients

The research related to human use complied with all the relevant national regulations and institutional policies in accordance with the tenets of the Helsinki Declaration and was approved by the Ethics Committee of the Fifth People’s Hospital of Suzhou. QFT-GIT assays and CT were performed for fast identification of tuberculosis, and all patients were tested for tuberculosis culture in the Department of Tuberculosis at the Fifth People’s Hospital of Suzhou from June 2016 to December 2020. The total number of tuberculosis patients in this experiment was 125 cases, all of whom were male, 108 were mycobacterium tuberculosis culture positive, 43 belonged to the normal tuberculosis infection (TB) group and 65 belonged to the tuberculosis complicated with silicosis (TB&SILs) group; 11 silicosis patients who were QFT-GIT negative belonged to the silicosis group (SILs), and 7 silicosis patients who were QFT-GIT positive but eliminated active pulmonary tuberculosis by CT belonged to the silicosis combined with latent tuberculosis infection group (SILs&LTBI). According to the result of QFT-GIT assays, the TB&SILs group was also divided into the QFT-GIT positive (TB&SILs-P, n = 38) and QFT-GIT negative (TB&SILs-N groups, n = 27). Before the QFT-GIT test, patients with HIV infection and the use of immunosuppressive agents were excluded.

### QuantiFERON-gold in-tube assay

QuantiFERON-TB Gold (QFT) Kit (QIAGEN, Shanghai, China) was used for Quantiferon-Gold-In tube assay. Blood samples were obtained after fasting and before 9 AM. Three heparin tubes were collected: (1) a negative-control tube (NIL tube), (2) an antigen tube (AG tube; containing a coating of specific M. tuberculosis antigens (ESAT-6, CFP-10, TB 7.7) that came into contact with the patient's T cells in the blood sample), and (3) a positive-control tube containing phytohaemagglutinin-P (PHA) (MIT tube) and incubate for 16–24 h at 37 °C. The concentration of IFN-γ secreted by the cells was measured by ELISA. The results were measured in pg/mL and interpreted in accordance with the manufacturer's recommendations as negative, positive, or indeterminate.

### Flow cytometric analysis

Monoclonal antibodies for CD8-APC (clone: HIT8a), CD56-PerCP(clone: 5.1H11) and PD-1 PE (clone: EH12.2H7) were purchased from Biolegend, CD4-PE-Texas Red (clone: S3.5) was purchased from eBioscience. 50 μl of fresh heparinized whole blood from the patients was incubated with the indicated antibodies (10 μL) for 15 min, lysed with FACSTM lysing solution (BD Biosciences, San Jose, CA, USA), washed with phosphate-buffered saline, fixed and eventually detected with a BD FACSAria with BD FACS Diva (BD Biosciences, San Jose, CA, USA) software support. The data were analysed using FlowJo software (Tree Star, Ashland, OR, USA).

### PD-1 antibodies blockad experiment

The PD-1 blocking antibody (Catalog#AF1086) was purchased from R&D company. PD-1 blocking antibody(10 μg/mL) or IgG (10 μg/mL) combine with PBS, tuberculosis antigens library (ESAT-6, CFP-10, TB 7.7) or PHA to stimulate the peripheral blood of patients with silicosis, and incubate for 16 to 24 h at 37 °C. The concentration of IFN-γ secreted by the cells was measured by ELISA.

### Data sharing

The datasets generated and analysed during the current study are available in the https://doi.org/10.6084/m9.figshare.21747248.v1 repository.

### Statistical analysis

All data were analyzed using Graph Pad Prism 5.0 software (GraphPad, San Diego, CA, USA) and are presented as the means ± standard deviations (SDs). The Mann‒Whitney U test and Student's t test were used for continuous variables such as the mean age, and the chi-square test was used to compare categorical variables (sensitivity). A two-tailed *p* < 0.05 was considered statistically significant.

## Results

### QFT-GIT was less sensitive in patients in the TB&SILs group than in the TB group

The background characteristics of 125 patients were shown in Table 1. There is no difference in age and gender in all groups. Using QFT-GIT to detect the patients in the TB group and TB&SILs group, the positive rate of QFT-GIT, which was 54.3% in the TB&SILs group, was significantly lower than that in the TB group (93.02%) (Table 1).

**Table 1:** Compare the positive rate of QFT-GIT between low TB group and TB&SILs group

	SILs group (n = 11)		SILs&LTBI group (n = 6)		TB group (n = 43)		TB&SILs group (n = 65)		*P* value
QFT-GIT positive (n, %)	0,	0	6,	100	40,	93.02	38,	58.46	< 0.0001
Tuberculosis cultures positive (n, %)	0,	0	0,	0	43,	100	65,	100	< 0.0001
Age (years)	66.18 ± 2.169		65.85 ± 2.774		68.20 ± 1.227		67.26 ± 1.002		0.3987
Male (n, %)	11,	100	6,	100	43,	100	65,	100	< 0.0001

*TB* pulmonary tuberculosis, *SILs* silicosis, *LTBI* latent tuberculosis infection

### No differences in the ratios of the peripheral blood lymphocyte subsets in the TB group, SILs group, SILs&LTBI group and TB&SILs group

**Fig. 1:**
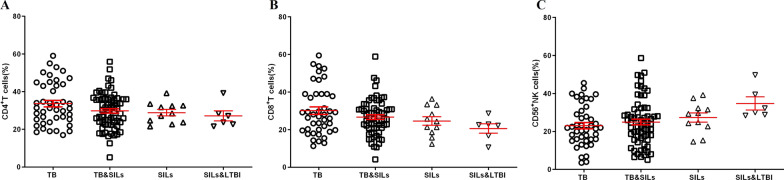
Differences in the proportions of peripheral blood CD4^+^T cells, CD8^+^T cells and CD56^+^NK lymphocyte subsets between the tuberculosis (TB) group, silicosis group (SILs), silicosis combined with latent tuberculosis infection group (SILs&LTBI) and pulmonary tuberculosis combined with silicosis (TB&SILs) group. **a** Statistical analysis of the proportions of CD4^+^T cells from the peripheral blood of patients in the TB group, SILs group, SILs&LTBI group and TB&SILs group; **b** statistical analysis of the proportions of CD8^+^T cells from the peripheral blood of patients in the TB group, SILs group, SILs&LTBI group and TB&SILs group; **c** statistical analysis of the proportions of CD56^+^T cells from the peripheral blood of patients in the TB group, SILs group, SILs&LTBI group and TB&SILs group *p < 0.05; **p < 0.01; ***p < 0.001

IFN-γ mainly comes from CD4^+^T cells CD8^+^T cells and NK cells, so this study further analyzed the expression of these lymphocyte subsets in peripheral blood of different patients. The results of flow cytometry showed that the proportions of CD4^+^T cells, CD8^+^T cells and CD56^+^NK cells in the TB group were (33.67 ± 1.168)%, (30.12 ± 1.916)% and (23.08 ± 1.650)%. The proportions of CD4^+^T cells, CD8^+^T cells and CD56^+^ NK cells in the TB&SILs group were (29.78 ± 1.190)%, (26.70 ± 1.195)% and (24.97 ± 1.544)%, respectively. Compared with the TB group, the proportion of CD4^+^T cells, CD8^+^T cells and CD56^+^NK in the TB&SILs group were no difference (*p* < 0.05) (Fig. 1).

### Silicosis had immune exhaustion state and PD-1 expression is negatively correlated with function of tuberculosis antigen-specific T cells

Immune exhaustion is an important factor in inhibiting T cell activation. PD-1 is an important molecule in T cell immune exhaustion, and its high expression can inhibit the expression of IFN-γ in CD4^+^T cells, CD8^+^T cells and CD56^+^NK cells.

**Fig. 2:**
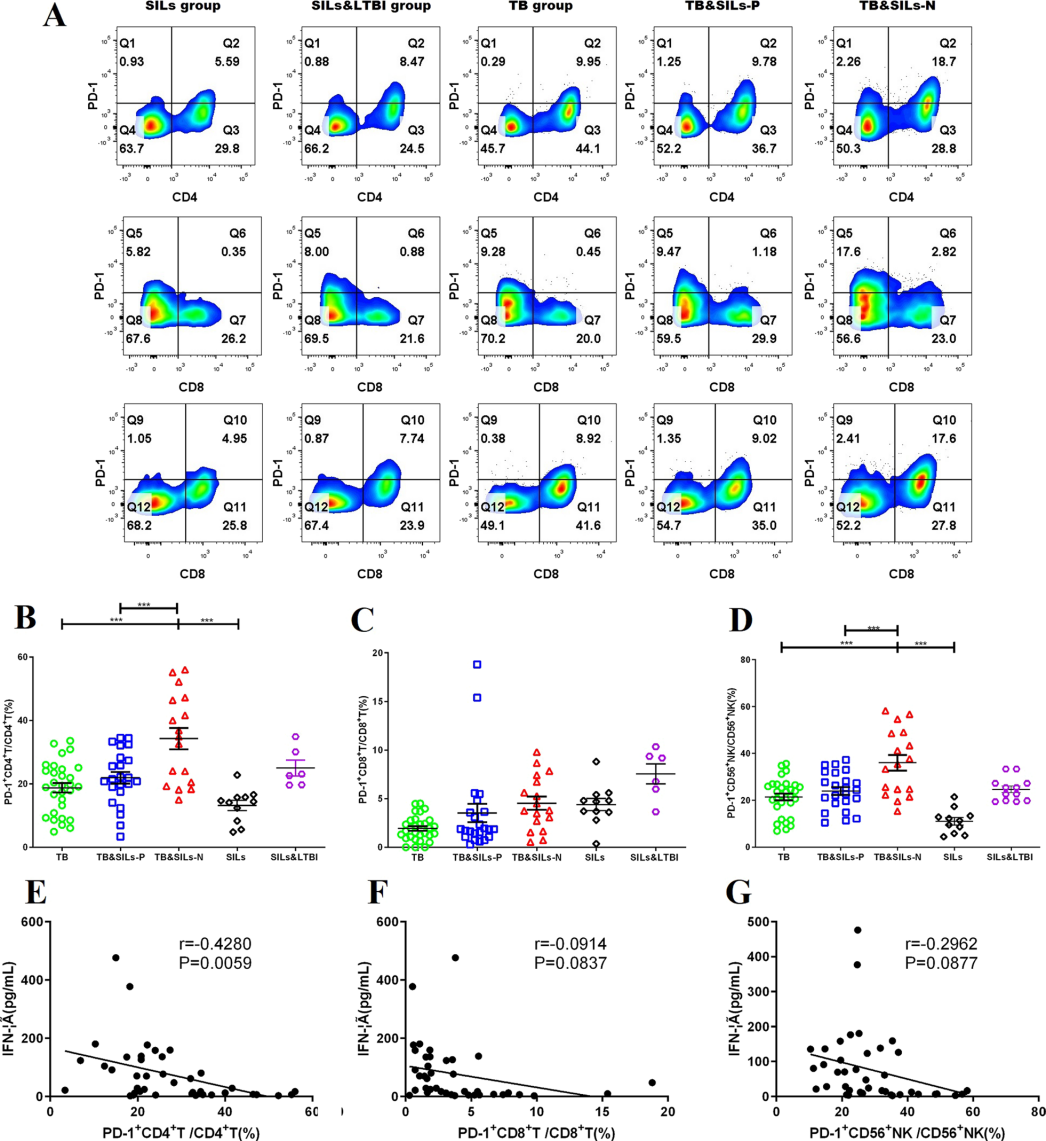
The expression of PD-1 on CD4^+^T cells CD8^+^T cells and CD56^+^NK cells in silicosis. **a** Gating strategy and expression profiles of PD-1 in peripheral CD4^+^T cells CD8^+^T cells and CD56^+^NK cells in TB group, SILs group, SILs&LTBI group TB&SILs-P group and TB&SILs-N group. **b** The expression of PD-1 on CD4^+^T cells in TB group, SILs group, SILs&LTBI group TB&SILs-P group and TB&SILs-N group; **c** The expression of PD-1 on CD8^+^T cells in TB group, SILs group, SILs&LTBI group TB&SILs-P group and TB&SILs-N group; **d** The expression of PD-1 on CD56^+^NK cells in TB group, SILs group, SILs&LTBI group TB&SILs-P group and TB&SILs-N group; **e** Analyzed the correlation between the level of IFN-γ and expression of PD-1 on CD4^+^T cells; **f** Analyzed the correlation between the level of IFN-γ and expression of PD-1 on CD8^+^T cells; **g** Analyzed the correlation between the level of IFN-γ and expression of PD-1 on CD56^+^NK cells. *p < 0.05; **p < 0.01; ***p < 0.001

Therefore, expression of PD-1 on the peripheral blood lymphocytes in TB group, SILs group, SILs&LTBI group and TB&SILs group were detected by flow cytometry in this study. Compared with the TB group and the SILs group the expression of PD-1 on CD4^+^T cells CD8^+^T cells and CD56^+^NK cells in the TB&SILs-N group was increased, and the differences were statistically significant (*P* < 0.01). Further comparisons revealed that the expression of PD-1 on CD4^+^T cells and CD56^+^NK cells in the TB&SILs-P group were also found to be higher than those in the TB&SILs-N (*P* < 0.001) (Fig. 2a–d).

Using tuberculosis antigens library (ESAT-6, CFP-10, TB 7.7) to stimulate the peripheral blood of patients with silicosis, and analyzed the correlation between the level of IFN-γ in the culture supernatant and PD-1 expression. The results show that PD-1 negatively associates with IFN-gamma but only significant for CD4^+^ T cells (Fig. 2e–g).

### PD-1 blocking antibody can increase sensitivity to QFT-GIT in silicosis

**Fig. 3:**
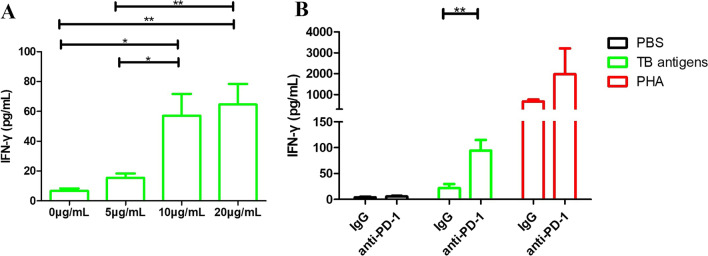
PD-1 blocking antibodies can increase sensitivity to QFT-GIT in silicosis. **a** Analyzed the optimal working concentration (0 μg/ml, 5 μg/ml, 10 μg/ml and 20 μg/ml,) of PD-1 blocking antibody; **b** analysis of the PD-1 blocking antibody effect on lymphocytes in silicosis. Black: lymphocytes stimulated with PBS, Green: lymphocytes stimulated with tuberculosis antigens library (ESAT-6, CFP-10, TB 7.7), Red: lymphocytes stimulated with PHA; *p < 0.05; **p < 0.01; ***p < 0.001

It has been reported that PD-1 blocking antibody can restore the IFN-γ expression of antigen-specific lymphocytes, so we used PD-1 blocking antibody to increase the sensitivity of QFT-GIT in silicosis. First we used dilution experiments to find the optimal working concentration of blocking antibody and found that 10 μg/mL antibody can increase the expression of IFN-γ of tuberculosis antigen T cells (Fig. 3a). Then we used PD-1 blocking antibody or IgG combine with PBS, tuberculosis antigens library (ESAT-6, CFP-10, TB 7.7) or PHA to stimulate the peripheral blood of patients with silicosis, and found blockading the PD-1 pathway can significantly restore IFN-γ expression of tuberculosis antigen-specific specific cells (Fig. 3b). Analyzed the QFT-GIT results of 7 TB&SILs-N patients after anti-PD-1 treatment and found that blocking the PD-1 signal pathway can improve the sensitivity of QFT (Table 2).

**Table 2:** Compare the positive rate of QFT-GIT between IgG treatment group and anti-PD-1 treatment group

	TB&SILs-N (n = 7)		TB&SILs-P (n = 4)		*P* value
QFT-GIT positive of IgG treatment group (n, %)	0,	0	4,	100	0.04
QFT-GIT positive of PD-1 treatment group (n, %)	6,	83.3	4,	100	

## Discussion

Silicosis is the most common type of pneumoconiosis [3]. It is caused by long-term inhalation of large amounts of free silica dust and is mainly characterized by extensive pulmonary nodular fibrosis. When severe, it affects respiratory function, and the patient loses the ability to work. Because silica damages phagocytic function, affects peripheral cells, and interferes with cytokine production, silica affects immune function and can easily predispose patients to tuberculosis [2, 8, 9]. The CT test of pneumoconiosis combined with pulmonary tuberculosis are more complicated, lacking typical clinical manifestations and being easily missed in clinical practice [10]. Existing studies have confirmed that TB-IGRA is the most sensitive test for the detection of patients with silicosis combined with pulmonary tuberculosis [11]. It can not only detect active tuberculosis but also prompt latent tuberculosis infection (LTBI) to provide an important basis for clinical treatment [5–7]. Early studies have confirmed that the false-negative rate of patients with low immunity (HIV infection) will increase significantly and other study also found that patients with silicosis have immunodeficiency [12, 13]. However, there is no study on whether this immunocompromised state will affect the sensitivity of TB-IGRA. Therefore, further analysis of the relationship between immune function and TB-IGRA will provide clinically more accurate test reports, which is conducive to more accurate diagnoses of tuberculosis.

The study found that the diagnostic efficiency of QFT-GIT in the patients from the TB&SILs group was significantly reduced but the proportion of CD4^+^T cells, CD8^+^T cells and CD56^+^NK in the TB&SILs group were no difference with TB group, SILs group and SILs&LTBI group.

Existing studies have found that PD-1/PD-L1 can inhibit the function of T cells [14, 15]. In the study of tumours and viruses, it has been found that high concentrations of antigens for a long time can induce CD4^+^T cells to express PD-1, decreases the IFN-γ expression of lymphocytes and lead to immune exhaustion [16, 17]. Some studies also found that silica instillation significantly elevated the expression of immune checkpoint molecules such as PD-1 in mice and suggested that silica is an important risk factor for T-cellcell exhaustion [18, 19]. Due to the negative correlation between PD-1 and IFN-γ and there is no study on T cell exhaustion in silicosis patients before, this study analysed the expression of PD-1 in silicosis and found that the peripheral blood lymphocytes of patients with silicosis highly expressed PD-1, and the PD-1 highly expression can inhibited the secretion of IFN-γ by lymphocytes (especially CD4^+^T cells). Further analysis found that the ratio of PD-1^+^CD4^+^T and IFN-γwere negatively correlated and blockaded the PD-1 pathway with antibodies can restore the sensitivity of QFT-GIT in silicosis.

Tuberculosis is the main cause of death in silicosis, and many studies have found that cytokines (IFN-γ, IL-1β and TNF-α) that can inhibit the immune escape of tuberculosis are decreased, but some Th2 cytokines are increased in silicosis [20, 21]. The main reason for the high incidence of tuberculosis in patients with silicosis is still unclear, and the methods for early diagnosis of pulmonary tuberculosis in silicosis patients are very limited. Research has indicated that higher expression of PD-1 inhibits the Th1 immune response, which suppresses the anti-TB immune response. This study found that the peripheral blood lymphocytes of patients with silicosis highly expressed PD-1, and the immune depletion of CD4^+^T cells and NK cells was the main reason for the higher false negative rate of QFT. The findings of this study suggest that patients with silicosis have immune exhaustion, which provides a new theory for the occurrence of silicosis combined with pulmonary tuberculosis. However, this study is not sufficiently systematic and in depth, and we analyzed the molecular mechanism of silicosis leading to a high incidence of tuberculosis and used animal models to explore the role of PD-1 in silicosis, which will provide new targets for the treatment and immune monitoring of silicosis.

## Conclusions

This is the first study to analyze the relationship between immune exhaustion and QFT-GIT in silicosis and found that the sensitivity of QFT-GIT was decreased by the expression of PD-1 on lymphocytes. Antibody blocking experiments increased the expression of IFN-γ and provided a new method to improve the sensitivity of QFT in silicosis. The study also found that silicosis can increase PD-1 expression, as PD-1 functions in infectious diseases, prompting immune exhaustion in silicosis, which may promote tuberculosis from latent to active infection. The study provided theoretical evidence for the diagnosis and immunotherapy of silicosis complications, and it has great value in clinical diagnostics and treatment.

## Data Availability

The datasets generated and analysed during the current study are available in the https://doi.org/10.6084/m9.figshare.21747248.v1.

## Abbreviations

TB-IGRA: IFN-γ release in vitro test
QFT-GIT: QuantiFERON-TB Gold
TB: Tuberculosis infection
TB&SILs: Tuberculosis complicated with silicosis
TB&SILs-P: Tuberculosis complicated with silicosis and QFT-GIT positive
TB&SILs-N: Tuberculosis complicated with silicosis and QFT-GIT negative
PHA: Phytohaemagglutinin-P
LTBI: Latent tuberculosis infection
